# Incidence and Risk of Cardiovascular Outcomes in Patients With Anorexia Nervosa

**DOI:** 10.1001/jamanetworkopen.2024.51094

**Published:** 2024-12-19

**Authors:** Mei-Chih Meg Tseng, Kuan-Rau Chiou, Joni Yu-Hsuan Shao, Hung-Yi Liu

**Affiliations:** 1 Department of Psychiatry, School of Medicine, College of Medicine, Taipei Medical University, Taipei, Taiwan; 2 Department of Psychiatry, Shuang Ho Hospital, Taipei Medical University, New Taipei City, Taiwan; 3 Department of Psychiatry, National Taiwan University College of Medicine, Taipei, Taiwan; 4 Divison of Cardiology, Department of Internal Medicine, Shuang Ho Hospital, Taipei Medical University, New Taipei City, Taiwan; 5 Graduate Institute of Biomedical Informatics, Taipei Medical University, Taipei, Taiwan; 6 Clinical Big Data Research Center, Taipei Medical University Hospital, Taipei, Taiwan; 7 Health Data Analytics and Statistics Center, Office of Data Science, Taipei Medical University, New Taipei City, Taiwan

## Abstract

**Importance:**

Anorexia nervosa (AN) is commonly associated with cardiovascular complications.

**Objective:**

To investigate the trajectories of the risk of cardiovascular conditions in a nationwide cohort of patients with AN in Taiwan.

**Design, Setting, and Participants:**

From a population-based health insurance database from January 1, 2011, to December 31, 2021, this longitudinal cohort study identified patients with AN and controls through propensity score matching at a 1:10 ratio according to sex, age, urbanization level of residence, socioeconomic status, and year of diagnosis. Data were analyzed from June 27, 2023, to February 23, 2024.

**Exposure:**

First-time diagnosis of AN by psychiatrists during the study period.

**Main Outcomes and Measures:**

Incidence and risk of composite cardiovascular conditions. Kaplan-Meier curves were used to estimate the cumulative incidence of major adverse cardiovascular events (MACE) and any cardiovascular condition. With adjustment for psychiatric comorbidities, conditional Cox proportional hazards regression analyses were performed to estimate the risk of cardiovascular events, which were presented as hazard ratios (HRs) and 95% CIs, relative to the comparison group. Risks of individual cardiovascular conditions were calculated during 3 follow-up periods after AN diagnosis.

**Results:**

The study population included 2081 patients with AN and 20 810 matched controls, for a total of 22 891 participants (mean [SD] age, 24.9 [9.9] years; 91.3% female). In total, 99 patients with AN (4.8%) had MACE vs 175 (0.8%) in controls, and 124 patients with AN (6.0%) had any cardiovascular condition vs 483 controls (2.3%). At the 5-year follow-up, the cumulative incidence rate of MACE was 4.82% (95% CI, 3.85%-6.02%) and of any cardiovascular condition was 6.19% (95% CI, 5.19%-7.53%). Compared with the control group, the AN group had significantly higher risks of MACE (adjusted HR [AHR], 3.78; 95% CI, 2.83-5.05) and any cardiovascular condition (AHR, 1.93; 95% CI, 1.54-2.41). The significantly increased risks of congestive heart failure, conduction disorder, and structural heart disease occurred in the initial follow-up period and disappeared after 60 months of follow-up. Notably, patients with AN did not have an increased risk of ischemic heart disease until after 60 months of follow-up (AHR, 3.01; 95% CI, 1.48-6.13).

**Conclusions and Relevance:**

In this national matched cohort study, increased risk of cardiovascular conditions was found in different periods after AN diagnosis. Clinicians should monitor comorbid cardiovascular conditions among patients with AN at initial presentation, during treatment, and at follow-up.

## Key Points

**Question:**

Do patients with anorexia nervosa (AN) have an increased risk of cardiovascular conditions?

**Findings:**

In this cohort study including 2081 patients with AN and 20 810 matched controls, the AN group had an increased rate and risk of cardiovascular outcomes. The increased risks of many cardiovascular conditions disappeared after 60 months of follow-up, whereas the risk of ischemic heart disease increased after 60 months.

**Meaning:**

These results suggest that clinicians should monitor comorbid cardiovascular conditions in patients with AN at different stages of treatment.

## Teaser

This cohort study examines the incidence and risk of cardiovascular outcomes and trajectories over time among patients with incident anorexia nervosa and a matched control group from a population-based health insurance database in Taiwan.

## Introduction

Anorexia nervosa (AN), an eating disorder, is characterized by an intense fear of weight gain and low body weight, and it is associated with various psychiatric and medical comorbidities.^1^ Although AN most commonly occurs in adolescents and young adults, individuals with AN have a 5- to 6-fold increased risk of mortality relative to their age-matched controls^2,3^ and a 2- to 3-fold increased risk of death due to cardiovascular causes.^2,4^ Cardiovascular abnormalities are common in patients with AN, with a rate of up to 87% in some stages of the illness.^5^ Marked weight loss and malnutrition in individuals with AN may lead to reduced left ventricular atrophy, mitral valve prolapse, bradycardia, hypotension, autonomic dysfunction, and peripheral vascular changes.^6^ Malnutrition associated with electrolyte disequilibrium or with refeeding in AN may be associated with more cardiovascular complications, including pericardial effusion,^7,8^ congestive heart failure,^9^ and QT interval prolongation.^6^ A few isolated case studies have reported acute myocardial infarction in the population with AN.^10,11^

Studies examining the cardiovascular complications associated with AN are mostly case studies or case-control studies with a small sample size, and they have only reported the frequency of such complications.^12,13^ Several systematic reviews and meta-analyses examining electrocardiographic findings,^14,15^ autonomic function test findings based on heart rate variability,^16^ or echocardiographic findings^17^ have demonstrated an increased risk of the aforementioned cardiovascular complications among patients with AN. Nonetheless, few epidemiological studies have examined the incidence and risk of cardiovascular outcomes in the AN population. To the best of our knowledge, only 2 register-based studies have been conducted.^18,19^ Only one of them reported the increased risk of cardiovascular events (a composite of ventricular tachycardia, aborted cardiac arrest, and cardiac arrest) in patients with AN, with a hazard ratio (HR) of 10.4 (95% CI, 2.6-41.6; *P* = .001), compared with the population-based cohort.^19^ However, the absolute numbers were small (n = 2), with a cumulative incidence of 0.5% in patients with AN and 0.07% in the population-based cohort (n = 3) after 10-year follow-up. More importantly, changes in the risk of individual cardiovascular conditions at follow-up over time are unknown.

Therefore, we examined the incidence and risk of cardiovascular outcomes in patients with incident AN and a matched control group by estimating HRs and cumulative incidence using data from a population-based health insurance database. Furthermore, we examined the trajectories of the incidence and risk of individual cardiovascular conditions over various follow-up periods.

## Methods

### Database Source

This study used data from the National Health Insurance Research Database (NHIRD), a claims-based database containing the information of the beneficiaries of Taiwan’s National Health Insurance (NHI) program. The NHI program covers 99.99% of Taiwan’s population (23 million), and it has service contracts with 93% of private and public hospitals and clinics.^20^ Situations in which a person would withdraw from Taiwan’s NHI program include death, missing for more than 6 months, and disqualification such as immigration and the expiration of the duration of stay of migrants.^21^ The percentage of disqualification is relatively minimal (around 2% of all insured individuals). The database was inaugurated in 1995 and is managed by the NHI Administration.^22^ NHIRD data are additionally linked to the Death Registry, to ascertain vital status and cause of death.^23^ Fifty reports have been published with validated findings for diagnosis codes for a wide range of health outcomes using the NHIRD,^24^ including cardiovascular diseases,^25,26,27^ stroke,^27,28,29,30,31,32^ types 1 and 2 diabetes,^33,34^ hypertension,^26,27,34^ hyperlipidemia,^26,34^ and psychiatric diseases.^34,35^ The study protocol was approved by the Taipei Medical University–Joint Institutional Review Board. Waiver of informed consent was granted because this study conducted a secondary analysis of existing data. The study followed the Strengthening the Reporting of Observational Studies in Epidemiology (STROBE) reporting guideline.

### Study Cohort

Data of individuals with AN diagnosed by psychiatrists from January 1, 2010, to December 31, 2021, were extracted from the NHIRD.^36^ The diagnosis of AN was based on the *International Classification of Diseases, Ninth Revision, Clinical Modification* (*ICD-9-CM*) code 307.1 (2010-2015) and the *ICD-10-CM* code F50.0 (2016-2021). Patients without any eating disorder during the same study period were selected as the comparison group. In this study, people who had missing data for age, sex, socioeconomic status, or urbanization level of residency were excluded. We also excluded patients who were younger than 10 years or who were 60 years or older in the AN cohort and their counterparts, leaving the age range in which AN is mostly diagnosed. To ensure that we included a newly diagnosed AN case, we excluded patients who had any AN diagnosis in 2010. The date of first diagnosis was considered the index date, and the index date was randomly assigned to the comparison group. We further excluded patients who had a diagnosis of a preexisting cardiovascular condition within 1 year prior to AN diagnosis and prior to the pseudo–index date in the comparison group. Propensity score matching was conducted using logistic regression to ensure comparability between individuals with AN and controls in a 1:10 matching ratio. Matching factors included sex, age, urbanization level of residence, socioeconomic status (SES), and year of medical visit, with a caliper distance set at less than 0.1. The logistic regression model estimated the propensity scores, which were then used to match individuals across the 2 groups. These variables are associated with both AN and cardiovascular disorders in the existing literature.^19,37,38^ The number of patients excluded in each step were present in the eFigure in Supplement 1.

The urbanization level of residence in Taiwan was defined according to the 2000 Taiwan census data from the Survey of Health Maintenance Organizations’ Current Status and Health Service Utilization, in which the urbanization level of the 368 towns in Taiwan is classified into 7 levels.^39^ Levels 1 and 2 represent urban areas, levels 3 and 4 represent suburban areas, and levels 5 to 7 represent rural areas. NHI enrollment is mainly through employment wage tax deduction for people with a well-defined monthly wage and through head-tax financing on farmers, fishermen, and people without a well-defined monthly wage. SES was defined according to the income information recorded in the Registry for Beneficiaries.^40^ The enrolled individuals were divided into 4 groups by SES (1 plus 2, 3, 4, and 5 plus 6), with groups 1 and 2 representing the highest SES and groups 5 and 6 the lowest SES.

### Outcome Measurement and Covariates

The primary outcomes were the occurrence of major adverse cardiovascular events (MACE) and any cardiovascular condition (congestive heart failure, stroke, ischemic heart diseases, conduction disorder, inflammatory heart disease, valve disease, cardiomyopathy, atherosclerosis, or cardiac arrest) according to their respective *ICD-9-CM* or *ICD-10-CM* codes (eTable 1 in Supplement 1). MACE was defined as presence of 1 or more discharge diagnosis of ischemic heart diseases, congestive heart failure, or stroke in any position, and all-cause death. Patients with cardiovascular conditions were defined as those with at least 2 outpatient diagnostic claims at a 28-day interval or 1 inpatient diagnostic claim after the index date.

The psychiatric comorbidities considered in the analysis included all psychiatric disorders except eating disorders (*ICD-9-CM* codes 290-319 [except 307] and *ICD-10-CM* codes F00-F99 [except F50]) (eTable 2 in Supplement 1). Psychiatric comorbidities have been postulated as risk factors for cardiovascular disease.^41^ The physical comorbidities included hypertension, hyperlipidemia, and diabetes (eTable 1 in Supplement 1). A patient was considered to have psychiatric or physical comorbidities if they had at least 2 outpatient diagnostic claims at a 28-day interval or 1 inpatient diagnostic claim in the year preceding the diagnosis of AN.

### Statistical Analysis

Data were analyzed from June 27, 2023, to February 23, 2024. Descriptive statistics (mean [SD], median [IQR], and frequency [percentage]) were calculated to describe the demographic and baseline characteristics of the AN and control groups. The standardized mean difference was calculated to examine the differences between the AN and control groups. The incidence of MACE and cardiovascular outcomes in patients with AN and controls were calculated. A Cox proportional hazards regression was performed to compute HRs and the corresponding 95% CIs to evaluate the risk of cardiovascular outcomes in AN group relative to the comparisons adjusting for psychiatric comorbidities and physical comorbidities. Plots of cumulative incidence function were generated, and the log-rank test was used to test the difference between 2 groups. The cumulative incidence and the corresponding 95% CIs were also calculated. To examine the risk of individual cardiovascular conditions during different periods, we estimated HRs for each cardiovascular condition over 3 follow-up periods (0-24, >24 to ≤60, and >60 months). All statistical analyses were performed using SAS, version 9.4 (SAS Institute Inc). Significance was indicated by 2-tailed *P* < .05 or by the 95% CIs in tests.

## Results

### Characteristics of Patients and Matched Controls

During the 11-year observation period, we identified 2081 patients with AN and 20 810 matched controls, for a total of 22 891 participants (mean [SD] age, 24.9 [9.9] years; 91.3% female and 8.7% male) (Table 1). The AN and control groups were balanced in terms of age, sex, SES, urbanization level of residence, and year of diagnosis after matching. The AN group predominantly comprised women (1899 [91.3%]), young people (1418 aged 10-29 years [72.9%]), individuals with the second highest SES (960 [46.1%]), and individuals who resided in highly urbanized cities (1362 [65.4%]). The mean (SD) duration of follow-up was 5.0 (3.3) years (median [IQR], 4.6 [2.1-7.8] years). The AN group had a significantly higher proportion of individuals with prior psychiatric comorbidities than the control group (715 [34.4%] vs 724 [3.5%]) (Table 1).

**Table 1.** Characteristics of Patients With AN and Matched Controls

Characteristic	Study group, No. (%)^a^		AN vs controls, SMD
	AN (n = 2081)	Matched controls (n = 20 810)	
Sex			
Male	182 (8.7)	1820 (8.7)	<0.001
Female	1899 (91.3)	18 990 (91.3)	<0.001
Age at diagnosis, y			
10-19	750 (36.0)	7500 (36.0)	<0.001
20-29	768 (36.9)	7680 (36.9)	<0.001
30-39	357 (17.2)	3570 (17.2)	<0.001
40-59	206 (9.9)	2060 (9.9)	<0.001
Socioeconomic status			
1 or 2 (Highest)	213 (10.2)	2130 (10.2)	<0.001
3	960 (46.1)	9600 (46.1)	<0.001
4	589 (28.3)	5890 (28.3)	<0.001
5 or 6 (Lowest)	319 (15.3)	3190 (15.3)	<0.001
Urbanization level of residence			
Urban	1362 (65.4)	13 620 (65.4)	<0.001
Suburban	586 (28.2)	5860 (28.2)	<0.001
Rural	133 (6.4)	1330 (6.4)	<0.001
Year of diagnosis			
2011-2013	523 (25.1)	5230 (25.1)	<0.001
2014-2016	484 (23.3)	4840 (23.3)	<0.001
2017-2019	599 (28.8)	5990 (28.8)	<0.001
2020-2021	475 (22.8)	4750 (22.8)	<0.001
Physical comorbidity			
Any	78 (3.7)	684 (3.3)	0.03
Hyperlipidemia	47 (2.3)	362 (1.7)	
Hypertension	18 (0.9)	393 (1.9)	
Diabetes	29 (1.4)	268 (1.3)	
Psychiatric comorbidity			
Any	715 (34.4)	724 (3.5)	0.86
Anxiety disorders	263 (12.6)	307 (1.5)	
Alcohol/drug use disorders	46 (2.2)	47 (0.2)	
Bipolar affective disorders	108 (5.2)	63 (0.3)	
Depressive disorders	449 (21.6)	240 (1.2)	
Other neurotic/adjustment disorders	129 (6.2)	98 (0.5)	
Schizophrenia	51 (2.5)	79 (0.4)	
Sleep disorders	231 (11.1)	403 (1.9)	

Abbreviations: AN, anorexia nervosa; SMD, standardized mean difference.

^a^ Ten controls without an eating disorder were matched to each patient with AN by age, sex, socioeconomic status, urbanization level of residence, and year of diagnosis.

### Incidence and Risk of Cardiovascular Outcomes in Individuals With AN

A total of 99 patients with AN (4.8%) had MACE vs 175 (0.8%) in controls; compared with the control group, the AN group had a higher incidence rate of MACE (9.63 [95% CI, 7.90-11.72] vs 1.65 [95% CI, 1.42-1.91] per 1000 person-years). In addition, 124 patients with AN (6.0%) had any cardiovascular condition vs 483 controls (2.3%), and the AN group had a higher incidence rate (12.55 [95% CI, 10.52-14.96] vs 4.60 [95% CI, 4.21-5.03] per 1000 person-years) (Table 2). Compared with the control group, the AN group had a significantly higher overall risk of MACE (adjusted HR [AHR], 3.78; 95% CI, 2.83-5.05) and any cardiovascular condition (AHR, 1.93; 95% CI, 1.54-2.41) than the control group. Conductive disorder was the most common cardiac condition in the AN group (Table 2). In adjusted models, patients with AN had a higher risk of congestive heart failure, conduction disorder, valvular disease, cardiomyopathy, and cardiac arrest compared with controls but not stroke, atherosclerosis, ischemic heart disease, and inflammatory heart disease (Table 2). The highest risks were observed in cardiac arrest (AHR, 34.08 [95% CI, 3.40-341.76]) and congestive heart failure (AHR, 4.64 [95% CI, 2.62-8.63]). Fewer than 3 people in the AN or control groups had inflammatory heart disease and fewer than 3 controls had cardiac arrest; therefore, the risk of these 2 conditions was not further analyzed during follow-up. Other rare diagnoses were grouped with diagnoses with similar pathogenesis or anatomy abnormalities, for example, atherosclerosis and stroke, were grouped into the cerebral and peripheral vascular disease category, and valvular disease and cardiomyopathy were grouped into structural heart disease category in the following analyses.

**Table 2.** Incidence and Risk of Cardiovascular Outcomes in Patients With AN in Taiwan, 2011 to 2021

Outcome by study group	No. of participants	Incidence rate per 1000 person-years (95% CI)	Crude HR (95% CI)	Adjusted HR (95% CI)^a^
Any MACE^b^				
AN	99	9.63 (7.90-11.72)	5.83 (4.56-7.46)	3.78 (2.83-5.05)
Controls	175	1.65 (1.42-1.91)	1 [Reference]	1 [Reference]
Congestive heart failure (inpatient)				
AN	12	1.16 (0.66-2.05)	4.74 (2.39-9.40)	5.07 (2.44-10.55)
Controls	26	0.24 (0.17-0.36)	1 [Reference]	1 [Reference]
Stroke (inpatient)				
AN	6	0.58 (0.26-1.29)	1.54 (0.65-3.63)	1.15 (0.44-2.97)
Controls	40	0.38 (0.28-0.51)	1 [Reference]	1 [Reference]
Ischemic heart disease (inpatient)				
AN	8	0.77 (0.39-1.55)	2.36 (1.09-5.09)	1.19 (0.50-2.84)
Controls	35	0.33 (0.24-0.46)	1 [Reference]	1 [Reference]
All-cause death				
AN	79	7.62 (6.11-9.50)	8.89 (6.58-12.02)	5.43 (3.80-7.76)
Controls	91	0.86 (0.70-1.05)	1 [Reference]	1 [Reference]
Any composite cardiovascular outcome^c^				
AN	124	12.55 (10.52-14.96)	2.72 (2.23-3.31)	1.93 (1.54-2.41)
Controls	483	4.60 (4.21-5.03)	1 [Reference]	1 [Reference]
Congestive heart failure				
AN	21	2.04 (1.33-3.13)	4.90 (2.92-8.25)	4.64 (2.62-8.23)
Controls	44	0.41 (0.31-0.56)	1 [Reference]	1 [Reference]
Stroke				
AN	15	1.45 (0.88-2.41)	2.11 (1.21-3.67)	1.31 (0.69-2.45)
Controls	73	0.69 (0.55-0.86)	1 [Reference]	1 [Reference]
Atherosclerosis				
AN	<3	0.10 (0.01-0.68)	1.71 (0.21-14.19)	1.29 (0.13-13.03)
Controls	6	0.06 (0.03-0.13)	1 [Reference]	1 [Reference]
Ischemic heart disease				
AN	26	2.52 (1.72-3.71)	2.10 (1.37-3.19)	1.34 (0.83-2.15)
Controls	128	1.21 (1.02-1.44)	1 [Reference]	1 [Reference]
Conduction disorder				
AN	53	5.23 (4.00-6.85)	2.69 (1.99-3.63)	1.74 (1.23-2.47)
Controls	206	1.95 (1.70-2.23)	1 [Reference]	1 [Reference]
Inflammatory heart disease				
AN	<3	0.19 (0.05-0.77)	10.19 (1.44-72.36)	8.37 (0.92-76.31)
Controls	<3	0.02 (0.01-0.08)	1 [Reference]	1 [Reference]
Valvular disease				
AN	29	2.83 (1.96-4.07)	2.02 (1.36-3.01)	1.56 (1.00-2.45)
Controls	148	1.40 (1.19-1.64)	1 [Reference]	1 [Reference]
Cardiomyopathy				
AN	3	0.29 (0.09-0.90)	3.84 (1.02-14.49)	4.13 (1.00-17.08)
Controls	8	0.08 (0.04-0.15)	1 [Reference]	1 [Reference]
Cardiac arrest				
AN	5	0.48 (0.20-1.16)	51.32 (6.00-439.31)	34.08 (3.40-341.76)
Controls	<3	0.01 (0.00-0.07)	1 [Reference]	1 [Reference]

Abbreviations: AN, anorexia nervosa; MACE, major adverse cardiovascular events; HR, hazard ratio.

^a^ Adjusted for psychiatric and physical comorbidities.

^b^ Includes patients with 1 or more discharge diagnosis and those with all-cause death.

^c^ Includes patients with 2 or more outpatient diagnoses or 1 or more discharge diagnosis.

### Cumulative Incidence of MACE and Composite Cardiovascular Conditions

**Figure 1.**
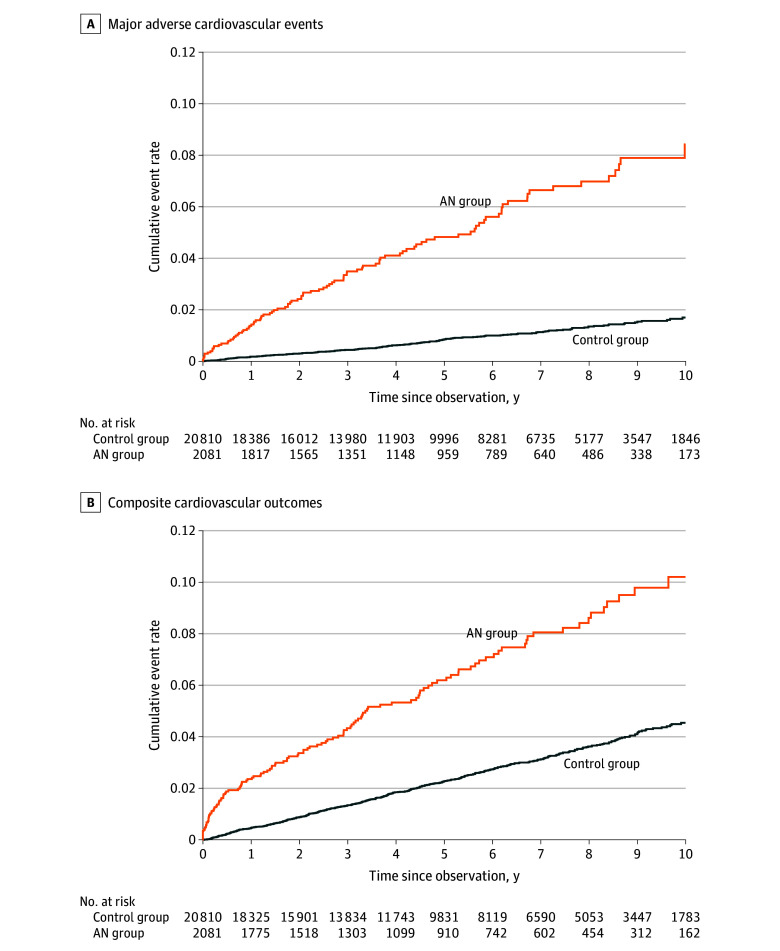
Cumulative Incidences of Major Adverse Cardiovascular Events and Composite Cardiovascular Outcome in Patients With Anorexia Nervosa (AN) and Controls

Figure 1 shows the cumulative incidence of MACE and cardiovascular outcomes. At the 5-year follow-up, the cumulative incidence rate of MACE was 4.82% (95% CI, 3.85%-6.02%) and of that of any cardiovascular condition was 6.19% (95% CI, 5.19%-7.53%) in the AN group, which were significantly higher than the incidence rates in the control group (0.85% [95% CI, 0.71%-1.01%] and 2.27% [95% CI, 2.04%-2.52%], respectively) (*P* < .001). The proportional hazards assumption was tested. Both models failed to reject the null hypothesis (*P* = .16 and *P* = .19, respectively).

### Risk of MACE and Composite Cardiovascular Conditions by Sex, Age, and Psychiatric Comorbidities

**Figure 2.**
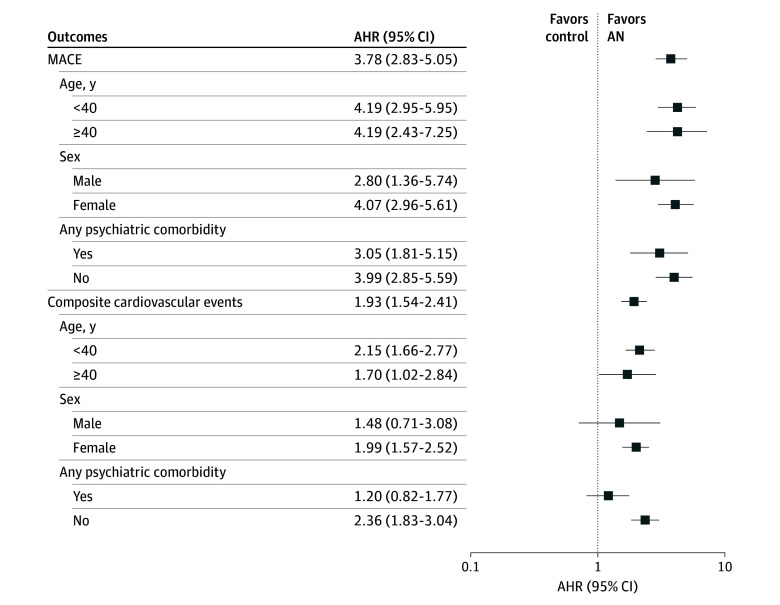
Adjusted Hazard Ratios (AHRs) for Major Adverse Cardiovascular Events (MACE) and Composite Cardiovascular Outcome by Age, Sex, and Psychiatric Comorbidity in Patients With Anorexia Nervosa (AN) and Controls

Within the AN group, older individuals (aged ≥40 years), men, and individuals with psychiatric comorbidities had a higher incidence of MACE. However, no significant difference was observed in the incidence of composite cardiovascular conditions between men and women in the AN group (eTable 3 in Supplement 1). Compared with their control counterparts, the AN subgroups also had a significantly higher risk of MACE when stratified by sex (eg, AHR for female patients, 4.07; 95% CI, 2.96-5.61), age (eg, AHR for ≥40 years, 4.19; 95% CI, 2.43-7.25), and psychiatric comorbidities (eg, AHR for none, 3.99; 95% CI, 2.85-5.59); in contrast to that, only the age-stratified AN subgroups had a significantly higher risk of composite cardiovascular conditions (eg, AHR for ≥40 years, 1.70; 95% CI, 1.02-2.84) (Figure 2 and eTable 3 in Supplement 1).

### Risk of Individual Cardiovascular Conditions During Follow-Up Periods

**Figure 3.**
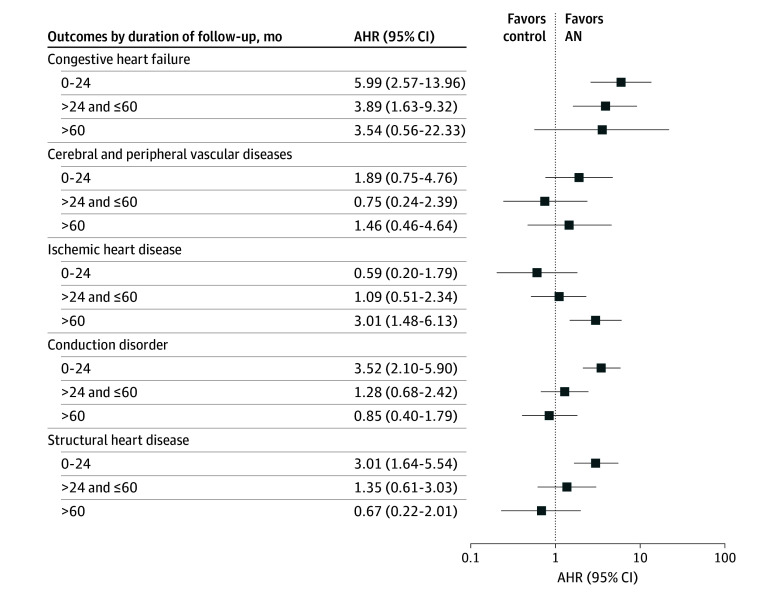
Risk of Individual Cardiovascular Conditions During Different Follow-Up Periods in Patients With Anorexia Nervosa (AN) and Controls Cerebral and peripheral vascular disease includes stroke and atherosclerosis, and structural heart disease includes valve disease and cardiomyopathy.

The increased risks of congestive heart failure, conductive disorder, and structural heart disease in the AN group occurred in the first 24 months after diagnosis and disappeared after more than 60 months of follow-up (Figure 3 and eTable 4 in Supplement 1). Notably, compared with the controls, patients with AN did not have an increased risk of ischemic heart disease during the initial 24-month period (AHR, 0.59; 95% CI, 0.20-1.79), but this risk increased only after 60 months of follow-up (AHR, 3.01; 95% CI, 1.48-6.13). For cardiovascular conditions, the pattern for the change in their incidence was parallel with that for their risk (eTable 4 in Supplement 1).

## Discussion

Our cohort study found that patients with AN had a nearly 4-fold higher risk of MACE and a nearly 2-fold higher risk of any cardiovascular condition than the matched controls. The cumulative 5-year incidences of MACE and composite cardiovascular outcomes were 4.82% and 6.19%, respectively. Our findings suggest that patients with AN have increased overall risks of cardiovascular conditions, including congestive heart failure, conductive disorder, structural heart disease, and cardiac arrest but not stroke, atherosclerosis, and ischemic heart disease. The risk of any cardiovascular condition increased in the initial 24-month follow-up and plateaued after 60 months. Notably, patients with AN did not have an increased risk of ischemic heart disease until after 60 months of follow-up.

Our study results confirm observations from previous studies^42^ that, compared with controls, cardiovascular conditions are common among patients with AN, but not ischemic heart disease. The commonly reported cardiovascular abnormalities among patients with AN are sinus bradycardia (range, 36%-95%),^43,44^ hypotension,^6^ and prolonged QTc interval (range, 0%-40%). Structural changes, such as pericardial effusion (range, 22%-35%),^7,45^ left ventricle mass reduction,^46^ myocardial fibrosis (25%),^47^ and mitral valve prolapse (range, 33%-60%),^48^ are also common. The true incidence of structural heart disease might be underestimated because not all patients admitted with eating disorders undergo echocardiography. Rather, these patients are more likely to have vital signs and electrocardiographic recordings, thus giving rise to a large body of evidence on bradycardia, hypotension, and electrical abnormalities.^49^ Our study found a higher cumulative incidence but a lower risk of MACE in the AN group relative to the control group compared with 1 prior registered study.^19^ This discrepancy might be due the definition of cardiovascular outcomes, sample characteristics, and length of follow-up.

Among the subgroups stratified by psychiatric comorbidities, a higher risk of cardiovascular conditions only existed among the AN group without psychiatric comorbidities compared with their control counterparts (eTable 3 in Supplement 1). This may be because people with mental illness already have an increased risk of cardiovascular events.^41,50^ The present study found that the incidence of cardiovascular conditions was higher among male and older patients with AN than among female and younger patients, respectively, which is consistent with the observation in another registered study.^18^ In that study, Kalla et al^18^ reported a significantly lower prevalence of coronary artery disease (4.4% vs 18.4%; *P* < .001) and congestive heart failure (2.1% vs 6.4%; *P* < .001) in patients with AN compared with the general population. By contrast, in our study, the AN group had had a 4-fold higher risk for congestive heart failure than the control group. Evidence shows that despite having a smaller left ventricular mass, most individuals, even those with severe AN, have normal left ventricular ejection fraction on echocardiography.^6^ Congestive heart failure may occur in patients with AN who have developed refeeding syndrome.^9,51,52^ We speculate that recognition and management of refeeding syndrome in severely malnourished patients with AN might decrease the incidence of congestive heart failure.

In this study, most cardiovascular conditions were in remission after 5 years except ischemic heart disease. This finding is corroborated by the recovery rate of 50% to 70% in patients with AN after 4 years of follow-up in a recent meta-analysis,^53^ and in previous studies,^45,54^ most of the cardiac complications improved with weight restoration. Similarly, genome-wide association studies did not support elevated cardiovascular risk in patients with AN due to shared genetic mechanisms between AN and cardiovascular diseases, but they suggested that cardiovascular diseases were a downstream consequence of AN.^55^

Studies have suggested that the mechanisms underlying coronary artery disease in patients with AN involve elevated total cholesterol levels during refeeding,^11^ endothelial dysfunction,^10^ or plaque characteristics.^10^ However, evidence for these candidate mechanisms are lacking. One study^56^ compared the intima-medial thickness of the carotid artery as measured by ultrasonography between patients with AN and controls and found no difference. Another study^57^ reported increased aortic pulse wave velocity, a marker of increased arterial stiffness, in the AN group. A recent study^58^ suggested that recovered patients with AN exhibit some adaptations in response to undernutrition (increased carotid artery stiffness, reduced aortic stiffness, vagal hyperactivity, and endothelial dysfunction), implying that these changes might have long-term consequences for cardiovascular health. Aging and the use of some psychotropic drugs might contribute to the risk of ischemic heart disease.^59^

### Limitations

This study has several limitations. First, cardiovascular outcomes were based on clinical diagnosis, and the validity of the AN or the AN subtype could not be confirmed in this study. Second, our patient cohort was selected from a population seeking medical treatment. Patients who received a diagnosis or treatment were likely to have more severe symptoms, which can lead to the overestimation of physical outcomes in our study. Third, we did not adjust for all potential confounders of cardiovascular events (eg, body weight, nutritional status, lifestyle, drug use, and family history) because these data were not available in the claims dataset. The number of events for several cardiovascular conditions were small, resulting in limited statistical power. Fourth, in cohorts with long follow-up periods, estimating the relevance of treatment and disease status for study outcomes can be difficult.^60^ Congestive heart failure can occur in malnourished patients with AN during treatment while refeeding. Due to the nature of a claims-based study, we do not know whether the cardiovascular event is a treatment-associated MACE. Finally, this study involved only individuals from a single ethnic group; therefore, the generalizability of the results may be limited.

## Conclusions

In this national matched cohort study, different cardiovascular abnormalities occurred in different periods after the diagnosis of AN, but many of them improved during follow-up. Clinicians should monitor comorbid cardiovascular conditions in patients with AN in different stages of treatment. The underlying mechanisms for the intermediate-term outcome of ischemic heart disease warrant further investigation.

## Notes

Supplement 1. **eTable 1.** *ICD-9-CM* and *ICD-10-CM* Codes for Cardiovascular Diagnosis and Physical Comorbidities **eTable 2.** *ICD-9-CM* and *ICD-10-CM* Codes for Psychiatric Disorders **eTable 3.** Stratified Analyses of Incidence and Risk of Cardiovascular Outcomes in Patients With Anorexia Nervosa **eTable 4.** Incidence and Risk of Individual Cardiovascular Conditions During Different Follow-Up Periods **eFigure.** Study Flow Diagram

Supplement 2.
Data Sharing Statement
