# Supplementary material for: Incidence and Risk of Cardiovascular Outcomes in Patients With Anorexia Nervosa
Source: JAMA Netw Open. 2024 Dec 19;7(12):e2451094. doi: 10.1001/jamanetworkopen.2024.51094 (PMC11659916; doi:10.1001/jamanetworkopen.2024.51094)
Supplement: Supplement 1. — eTable 1. ICD-9-CM and ICD-10-CM Codes for Cardiovascular Diagnosis and Physical Comorbidities eTable 2. ICD-9-CM and ICD-10-CM Codes for Psychiatric Disorders eTable 3. Stratified Analyses of Incidence and Risk of Cardiovascular Outcomes in Patients With Anorexia Nervosa eTable 4. Incidence and Risk of Individual Cardiovascular Conditions During Different Follow-Up Periods eFigure. Study Flow Diagram [file jamanetwopen-e2451094-s001.pdf]

Supplementary Online Content

Tseng M-CM, Chiou K-R, Shao JY-H, Liu H-Y. Incidence and risk of cardiovascular outcomes in patients with anorexia nervosa in Taiwan. *JAMA Netw Open*. 2024;7(12):e2451094. doi:10.1001/jamanetworkopen.2024.51094

eTable 1. *ICD-9-CM* and *ICD-10-CM* Codes for Cardiovascular Diagnosis and Physical Comorbidities

eTable 2. *ICD-9-CM* and *ICD-10-CM* Codes for Psychiatric Disorders

eTable 3. Stratified Analyses of Incidence and Risk of Cardiovascular Outcomes in Patients With Anorexia Nervosa

eTable 4. Incidence and Risk of Individual Cardiovascular Conditions During Different Follow-Up Periods

eFigure. Study Flow Diagram

This supplementary material has been provided by the authors to give readers additional information about their work.

© 2024 Tseng M-CM et al. *JAMA Network Open*

eTable 1. *ICD-9-CM* and *ICD-10-CM* Codes for Cardiovascular Diagnosis and Physical Comorbidities

Cardiovascular diagnosis	ICD-9-CM (2010–2015)	ICD-10-CM (2016–2021)
Congestive heart failure	428	I50
Stroke	430–438	I60–I69, G46
Atherosclerosis	440	I70
Ischemic heart disease	410–414	I20–I22, I24–I25
Conduction disorder	426.0, 426.1, 426.7, 427.0–1	I44–I45, I47–I49
Inflammatory heart disease	421.0, 422.9	I33, I40
Valve disorders	424.0. 424.1,	I34–I35, I39
Cardiomyopathy	425.1, 425.4, 425.5, 425.7	I42 (excluding I42.3–I42.4, I42.7–I42.8), I43
Cardiac arrest	427.5	I46
Physical comorbidities		
Hypertension	401-405	I10-I16
Hyperlipidemia	272	E71.30, E75.21, E75.22, E75.24, E75.3, E75.5, E75.6, E77, E78.0-E78.6, E78.70, E78.8, E78.9, E08-E11, E13, E88.1, E88.2, E88.89
Diabetes mellitus	250	E08-E11, E13

Abbreviations: ICD-9-CM and ICD-10-CM: International Classification of Diseases, Ninth and Tenth Revisions, Clinical Modification.

© 2024 Tseng M-CM et al. *JAMA Network Open*

eTable 2. *ICD-9-CM* and *ICD-10-CM* Codes for Psychiatric Disorders

	ICD-9-CM (2010–2015)	ICD-10-CM (2016–2021)
Anxiety disorders	300.0, 300.2	F40–F41
Alcohol/drug use disorders	291–292, 303–305	F10–F19, F55
Bipolar affective disorders	296 (excluding 296.2–296.3, 296.99), 298.1, 301.1x (excluding 301.12)	F30–F31, F34 (excluding F34.1)
Depressive disorders	296.2–296.3, 296.82, 298.0,301.12, 309.0, 309.1, 311, 300.4	F32.0, F33, F34.1, F43.21
Other neurotic/adjustment disorders	300 (excluding 300.0, 300.2, and 300.4), 308, 309 (excluding 309.0, 309.1), 312–313	F42–F45 (excluding F43.21), F48.1, F48.9, F68, F93.0, F94.0, F99
Schizophrenia	295, 297, 298.2-298.9, 301.0, 301.2	F20-F25, F28–F29, F60.0–F60.1
Sleep disorders	307.4, 780.5	F51, G47.0–G47.3, G47.5, G47.61, G47.69, G47.8–G47.9

Abbreviation: *ICD-9-CM* and *ICD-10-CM*: *International Classification of Diseases, Ninth and Tenth Revisions, Clinical Modification*.

© 2024 Tseng M-CM et al. *JAMA Network Open*

eTable 3. Stratified Analyses of Incidence and Risk of Cardiovascular Outcomes in Patients With Anorexia Nervosa

	AN			Controls			AN vs. Controls
	N	R (95% CI)	*P*	N	R (95% CI)	*P*	aHR (95% CI)
MACE[a]	99	9.63 (7.90–11.72)		175	1.65 (1.42–1.91)		3.78 (2.83–5.05)
Age			<.001			<.001	
<40	68	7.23 (5.70–9.17)		112	1.16 (0.97–1.40)		4.19 (2.95–5.95)
≥40	31	35.41 (24.90–50.35)		63	6.44 (5.03–8.24)		4.19 (2.43–7.25)
Sex			.05			<.001	
Males	14	14.93 (8.84–25.21)		42	4.27 (3.15–5.77)		2.80 (1.36–5.74)
Females	85	9.09 (7.35–11.25)		133	1.38 (1.17–1.64)		4.07 (2.96–5.61)
Psychiatric comorbidity			<.001			<.001	
Yes	56	17.66 (13.59–22.94)		19	6.20 (3.95–9.72)		3.05[c] (1.81–5.15)
No	43	6.05 (4.48–8.15)		156	1.51 (1.29–1.77)		3.99[c] (2.85–5.59)
Composite CV outcome[b]	124	12.55 (10.52–14.96)		483	4.60 (4.21–5.03)		1.93 (1.54–2.41)
Age			<.001			<.001	
<40	99	10.91 (8.96–13.29)		346	3.62 (3.26–4.03)		2.15 (1.66–2.77)
≥40	25	30.95 (20.91–45.80)		137	14.51 (12.27–17.15)		1.70 (1.02–2.84)
Sex			.96			.001	
Males	11	12.19 (6.75–22.01)		62	6.39 (4.98–8.20)		1.48 (0.71–3.08)
Females	113	12.58 (10.47–15.13)		421	4.42 (4.02–4.8)7		1.99 (1.57–2.52)
Psychiatric comorbidity			.02			<.001	
Yes	55	18.28 (14.03–23.81)		50	16.97 (12.86–22.38)		1.20[c] (0.82–1.77)
No	69	10.04 (7.93–12.71)		433	4.25 (3.87–4.67)		2.36[c] (1.83–3.04)

Abbreviations: AN: anorexia nervosa; MACE: major adverse cardiovascular event; CI: confidence interval; CV: cardiovascular; aHR: adjusted hazard ratio; N: number of events; R: indicates incidence rate per 1000 person-years; *P*: indicates comparison within AN or control group

a: include patients with one or more discharge diagnosis of ischemic heart diseases, congestive heart failure, and stroke in any position, and those with all-cause death;

b: include patients with two or more outpatient diagnoses or one or more discharge diagnosis of atherosclerosis, cardiac arrest, cardiomyopathy, conduction disorders, congestive heart failure, inflammatory heart diseases, ischemic heart diseases, stroke, and valvular diseases in any position.

c: adjusted HR for physical comorbidities only

aHR: adjusted for psychiatric comorbidities and physical comorbidities

© 2024 Tseng M-CM et al. *JAMA Network Open*

eTable 4. Incidence and Risk of Individual Cardiovascular Conditions During Different Follow-Up Periods

CV outcome	0–24 months			>24 and ≤60 months			> 60 months		
	N	R (95% CI)	aHR (95% CI)	N	R (95% CI)	aHR (95% CI)	N	R (95% CI)	aHR (95% CI)
Congestive heart failure									
AN	11	3.04 (1.68–5.49)	5.99 (2.57–13.96)	8	2.13 (1.06–4.26)	3.89 (1.63–9.32)	<3	0.69 (0.17–2.75)	3.54 (0.56–22.33)
Controls	17	0.46 (0.29–0.75)	1	22	0.57 (0.37–0.86)	1	5	0.16 (0.07–0.39)	1
Cerebral and peripheral vascular diseases									
AN	7	1.93 (0.92–4.05)	1.89 (0.75–4.76)	4	1.06 (0.40–2.83)	0.75 (0.24–2.39)	5	1.70 (0.71–4.09)	1.46 (0.46–4.64)
Controls	30	0.82 (0.57–1.17)	1	29	0.75 (0.52–1.07)	1	20	0.65 (0.42–1.01)	1
Ischemic heart disease									
AN	4	1.10 (0.41–2.94)	0.59 (0.20–1.79)	10	2.65 (1.43–4.93)	1.09 (0.51–2.34)	12	4.13 (2.35–7.27)	3.01 (1.48–6.13)
Controls	41	1.12 (0.82–1.52)	1	48	1.24 (0.93–1.64)	1	39	1.28 (0.93–1.75)	1
Conduction disorder									
AN	28	7.81 (5.39–11.31)	3.52 (2.10–5.90)	15	4.05 (2.44–6.71)	1.28 (0.68–2.42)	10	3.52 (1.89–6.54)	0.85 (0.40–1.79)
Controls	62	1.69 (1.32–2.17)	1	73	1.88 (1.50–2.37)	1	71	2.34 (1.85–2.95)	1
Structural heart disease									
AN	20	5.55 (3.58–8.60)	3.01 (1.64–5.54)	8	2.14 (1.07–4.28)	1.35 (0.61–3.03)	4	1.37 (0.52–3.66)	0.67 (0.22–2.01)
Controls	48	1.31 (0.99–1.74)	1	57	1.47 (1.13–1.90)	1	51	1.67 (1.27–2.20)	1

Abbreviation: CV: cardiovascular; AN: anorexia nervosa; aHR: adjusted hazard ratio;
R: indicated incidence rate per 1000 person-years;
Inflammatory heart disease and cardiac arrest were not analyzed due to an overly small sample size.
Cerebral/peripheral vascular disease include stroke and atherosclerosis, and structural heart disease include valve disease and cardiomyopathy.
aHR: adjusted for psychiatric comorbidities

© 2024 Tseng M-CM et al. *JAMA Network Open*

eFigure. Study Flow Diagram

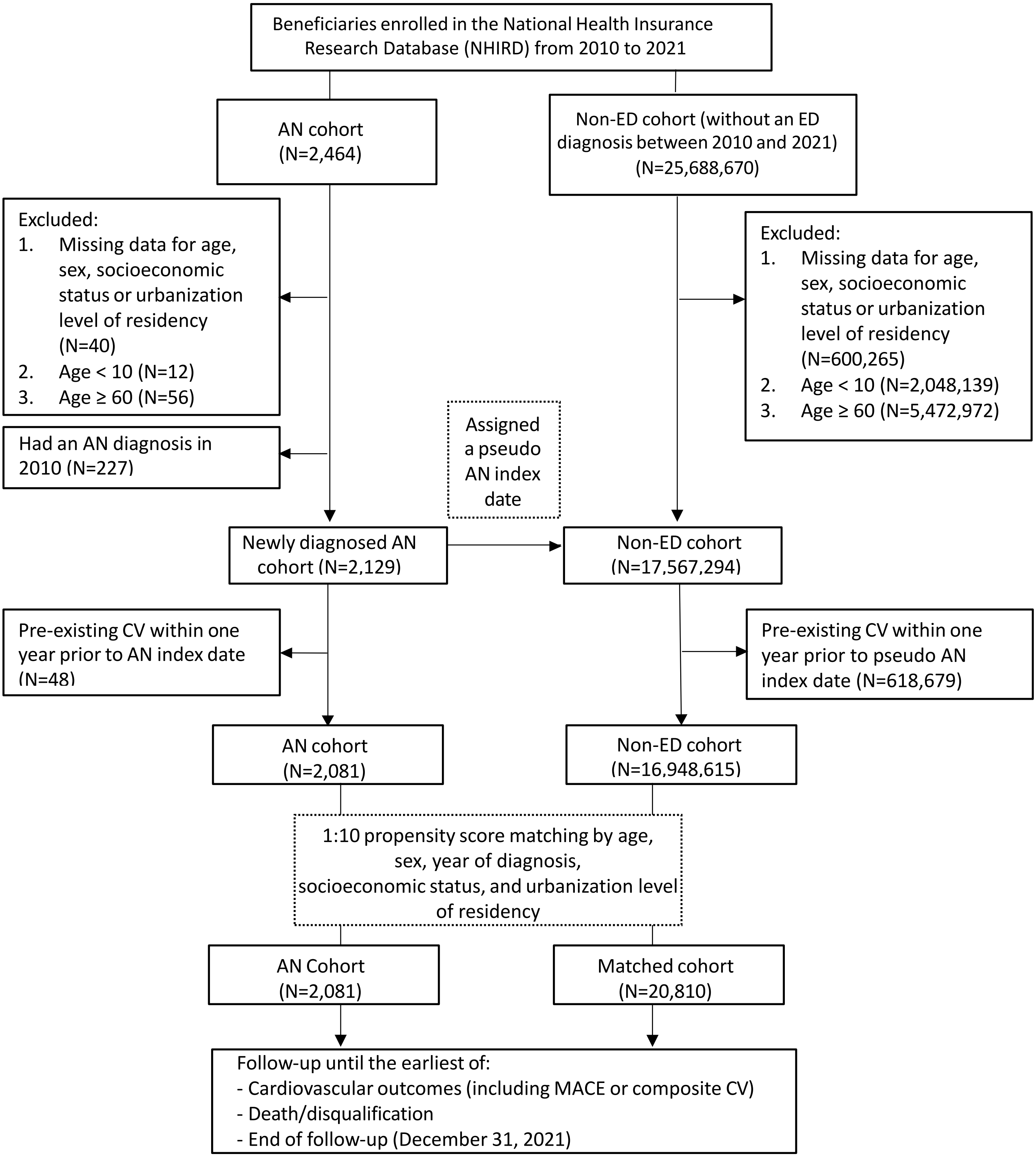

© 2024 Tseng M-CM et al. *JAMA Network Open*
