# Supplementary material for: Incidence and Risk of Cardiovascular Outcomes in Patients With Anorexia Nervosa
Source: JAMA Netw Open. 2024 Dec 19;7(12):e2451094. doi: 10.1001/jamanetworkopen.2024.51094 (PMC11659916; doi:10.1001/jamanetworkopen.2024.51094)
Supplement: Supplement 2. — Data Sharing Statement [file jamanetwopen-e2451094-s002.pdf]

Data Sharing Statement

Tseng. Incidence and Risk of Cardiovascular Outcomes in Patients with Anorexia Nervosa in Taiwan. *JAMA Netw Open*. Published December 19, 2024.
doi:10.1001/jamanetworkopen.2024.51094

Data
Data available: No

Additional Information
Explanation for why data not available: The data supporting the findings of this study are available at the Health and Welfare Data Science Center of the Ministry of Health and Welfare of Taiwan. Restrictions apply to the availability and analysis of these data.
